# WHO awards countries for progress in eliminating industrially produced trans fats for first time

**Published:** 2024-03

**29 January 2024** - WHO has awarded its first-ever certificates validating progress in eliminating industrially produced trans fatty-acids to five countries. Denmark, Lithuania, Poland, Saudi Arabia, and Thailand have each demonstrated they have a best practice policy for industrially produced trans-fatty acids (iTFA) elimination in effect, supported by adequate monitoring and enforcement systems. WHO also released results from the first five years of its REPLACE initiative to eliminate iTFA.

While the ambitious target set by WHO in 2018—to fully eliminate iTFA from the global food supply by the end of 2023—was not met, there has been remarkable progress made towards this goal in every region of the world. In 2023 alone, new best-practice policies became effective in 7 countries (Egypt, Mexico, Moldova, Nigeria, North Macedonia, Philippines, and Ukraine).

Trans-fatty acids (TFA) are semisolid to solid fats that occur in two forms: industrially produced and naturally occurring. Intake of TFA is associated with increased risk of heart attacks and death from heart disease. TFA have no known health benefits, and foods high in iTFA (e.g. fried foods, cakes and ready meals) are often high in sugar, fat and salt.

A total of 53 countries have now best practice policies in effect for tackling iTFA in food, vastly improving the food environment for 3.7 billion people, or 46% of the world’s population, as compared to 6% just 5 years ago. These policies are expected to save approximately 183 000 lives a year.

“Trans fat has no known health benefit, but huge health risks,” said Dr Tedros Adhanom Ghebreyesus, WHO Director-General. “We are very pleased that so many countries have introduced policies banning or limiting trans fat in food. But introducing a policy is one thing; implementing it is another. I congratulate Denmark, Lithuania, Poland, Saudi Arabia and Thailand, who are leading the world in monitoring and enforcing their trans fat policies. We urge other countries to follow their lead.”

Accelerating efforts to achieving best-practice policies in just 8 countries with the highest needs would eliminate 90% of the global iTFA burden, representing a unique opportunity to see in our lifetime a world free from deaths attributable to iTFA.

The WHO validation programme for iTFA elimination recognizes those countries which went beyond introducing best practice policies by ensuring rigorous monitoring and enforcement systems in place. Monitoring and enforcing compliance with policies is critical to maximizing and sustaining the health benefits of iTFA elimination.

Best practices in iTFA elimination policies follow WHO criteria and limit iTFA use in all settings. There are two best-practice policy options: 1) mandatory national limit of 2 grams of iTFA per 100 grams of total fat in all foods; and 2) mandatory national ban on the production or use of partially hydrogenated oils (a major source of trans fat) as an ingredient in all foods. For some countries, an optimal programme would implement both policies, due to the sources of trans fat.

“Trans fat elimination is economically, politically, and technically feasible and saves lives at virtually no cost to governments or consumers. This harmful compound is unnecessary, and no one misses it when it’s gone,” said Dr Tom Frieden, President and CEO of Resolve to Save Lives. “We are winning the battle against trans fat, but countries without regulations are at risk of becoming dumping grounds for TFA products. Governments and the food industry have a responsibility to ensure that doesn’t happen.”

WHO also encourages food manufacturers—the producers of raw materials and final food products—to eliminate iTFA from their products. The food industry has made good progress so far, as presented in a November 2023 WHO report.

Despite recent successes in eliminating iTFA from food globally, over half of the world’s population remain unprotected from its harmful impacts, thus putting them at a potential risk of increasing heart disease.

While countries should continue to strive for total elimination of iTFA, based on what has been achieved in the 5 years since the global call for elimination, WHO proposes a revised new target for virtual elimination of iTFA globally by 2025. The target includes:

- best-practice elimination policies are passed in countries that account for at least 90% of the total global iTFA burden.
- best practice policies are passed in countries that account for at least 70% of the total burden within regions.

Eliminating iTFA is a powerful way to prevent heart disease and the high costs to individuals and economies in medical treatment and lost productivity. WHO remains committed to supporting countries in their efforts and celebrating their achievements.

The next application cycle for the iTFA elimination validation programme will open in March 2024 and applications will be received on a continued basis.

Available from: https://www.who.int/news/item/29-01-2024-who-awards-countries-for-progress-in-eliminating-industrially-produced-trans-fats-for-first-time

